# Material Removal Mechanisms of Polycrystalline Silicon Carbide Ceramic Cut by a Diamond Wire Saw

**DOI:** 10.3390/ma17174238

**Published:** 2024-08-27

**Authors:** Huyi Yang, Ming Fu, Xin Zhang, Kailin Zhu, Lei Cao, Chunfeng Hu

**Affiliations:** 1 Nuclear Power Institute of China, Chengdu 610213, China; huygens2021@163.com (H.Y.); fm7887077@163.com (M.F.); 17828195929@163.com (X.Z.); 13548003532@163.com (K.Z.); 2 Key Laboratory of Advanced Technologies of Materials, Ministry of Education, School of Materials Science and Engineering, Southwest Jiaotong University, Chengdu 610031, China

**Keywords:** polycrystalline SiC, diamond wire cutting, feed rate, removal mechanism

## Abstract

Polycrystalline silicon carbide (SiC) is a highly valuable material with crucial applications across various industries. Despite its benefits, processing this brittle material efficiently and with high quality presents significant challenges. A thorough understanding of the mechanisms involved in processing and removing SiC is essential for optimizing its production. In this study, we investigated the sawing characteristics and material removal mechanisms of polycrystalline silicon carbide (SiC) ceramic using a diamond wire saw. Experiments were conducted with high wire speeds of 30 m/s and a maximum feed rate of 2.0 mm/min. The coarseness value (*R*_a_) increased slightly with the feed rate. Changes in the diamond wire during the grinding process and their effects on the grinding surface were analyzed using scanning electron microscopy (SEM), laser confocal microscopy, and focused ion beam (FIB)-transmission electron microscopy (TEM). The findings provide insights into the grinding mechanisms. The presence of ductile grinding zones and brittle fracture areas on the ground surface reveals that external forces induce dislocation and amorphization within the grain structure, which are key factors in material removal during grinding.

## 1. Introduction

The applications of silicon carbide (SiC) materials have significantly expanded recently due to their superior mechanical and physical properties [1,2]. These materials are characterized by high strength, corrosion resistance, low thermal expansion, high thermal conductivity, excellent tribological properties, and semi-conductive nature [3,4]. Consequently, SiC is increasingly used in diverse fields such as defense, electrical and electronics technologies, the automotive industry, aerospace, and biomedical applications [5,6]. Despite these advantages, achieving high-precision and high-efficiency machining of silicon carbide remains challenging. This difficulty arises from the tendency of micro-cracks to form on the surface or subsurface of the material during machining, which can degrade surface finish, dimensional accuracy, and material strength [7,8,9].

The processing of silicon carbide ceramics primarily relies on diamond grinding, with the removal mechanisms being categorized into brittle fracture removal and plastic deformation removal [10,11,12,13]. In brittle removal, the material experiences forces that exceed its fracture toughness, causing cracks to propagate rapidly, and the material is removed in a fragmented manner. This mode of removal often leads to degradation in the surface and subsurface quality of the material, and it may trigger more severe crack propagation or failure. Conversely, plastic removal involves the gradual removal of material through plastic deformation mechanisms such as slip, twinning, or dislocation motion. When diamond grinding is applied to brittle materials, the primary removal mechanism is brittle fracture, which is typically accompanied by a degree of plastic deformation. Consequently, the workpieces usually require additional polishing to mitigate the performance degradation caused by microcracks introduced during the grinding process. Polycrystalline silicon carbide ceramics face similar challenges in processing. As a material with significant engineering applications, the fundamental goal of efficient grinding is to maximize material removal rates while maintaining sufficient surface integrity and dimensional accuracy. Therefore, a comprehensive understanding of the ceramic grinding process is crucial.

To optimize the grinding process of silicon carbide, an in-depth study of its removal mechanism is essential. Currently, two main methods are used to study this mechanism: experimental research and simulation analysis. For experimental studies, single-point diamond scratching and single-point diamond turning (SPDT) techniques are mainly used [14,15,16]. These methods provide intuitive data on the grinding process; however, they differ from the multi-grain and polycrystalline silicon carbide grinding conditions in real industrial environments, which limits the general applicability of the results. For simulation analysis, the main reliance is on Molecular Dynamics (MD) simulation [17,18], which can model grinding processes under different conditions but is usually based on idealized models. For example, during the actual grinding process, the abrasive grains undergo wear and significant changes in their shape, which are often ignored in MD simulations.

Wire saws have become the primary tool for cutting semiconductors, crystals, various single crystals, oxide semiconductors, magnetic materials, and other brittle materials. Wang et al. studied the impact of cutting fluid on the grinding quality of silicon carbide (SiC) using a wire saw. Their results showed that with a feed rate of 0.25 mm/min and a wire speed of 1.8 m/s, a cutting fluid containing 0.2 wt% nano-SiC improved the grinding quality [19]. Hsu et al. employed an abrasive-free ultrasonic wire saw for processing Al_2_O_3_ [20]. Their findings indicated that ultrasonic processing is a viable method, achieving surface quality comparable to that of wire sawing. Harin et al. explored the effects of abrasive wire usage on machining, noting that while the impact of abrasive grain morphology and its mechanisms require further study, their research provided significant insights into the machining process [21]. Huang et al. used a diamond wire saw to investigate the effects of feed rate, wire speed, and wire length on surface quality [22]. Their study found that slower wire speeds removal by brittle fracture, whereas faster speeds tended to produce grooves on the surface. Cvetkovic et al. compared dicing and wire saw techniques for grinding polycrystalline and single-crystal silicon carbide [23]. Their experiments demonstrated that wire sawing has advantages for curved surface grinding. Zhang et al. examined the surface quality of Si_3_N_4_ using a diamond wire saw at a wire speed of 1600 m/s and a feed rate of 0.2 mm/min [24]. They discovered that higher wire speeds were effective in reducing the Ra value of the surface. Previous research has provided valuable insights into wire saw processing of brittle materials, but some limitations remain. Specifically, studies on removing polycrystalline silicon carbide (SiC) have focused on low wire speeds and feed rates (0.25 mm/min), which have not significantly improved removal efficiency. Additionally, the microscopic mechanisms of wire saw removal are not yet well understood. To address these gaps, this paper will employ high wire speeds and feed rates, along with FIB-TEM techniques, to conduct a detailed analysis of the removal mechanisms of wire sawing [25,26,27].

In this paper, we investigate the sawing characteristics and material removal mechanisms of polycrystalline silicon carbide using a diamond wire. The sawing tests were carried out on a reciprocating line with a fixed-plated diamond wire in the sawing machine at line speeds up to 30 m/s and a maximum feed rate of 2.0 mm/min. We analyzed in detail the changes in the diamond wire during the grinding process and its effect on the grinding surface. In particular, we analyzed the grinding surface quality under high-speed wire grinding conditions using SEM and laser confocal microscopy. Finally, by using the ion beam cutting technique (FIB), we intercepted the internal structure of silicon carbide usually in the grinding direction, and used TEM to analyze the mechanism of diamond wire removal on polycrystalline materials.

## 2. Experimental Procedure

The lab-made SiC powders were used to sinter silicon carbide (SiC) ceramic. In order to obtain a uniform distribution of fine particles, the powders were milled in a plastic bottle drily with agate balls for 10 h. The dried powder was placed in a carbon mold and sintered by the hot press (ZT-40-21YT, Chenhua Technology Co., Ltd., Shanghai, China) at 1850 °C for 2 h under a pressure of 30 MPa in argon. The mechanical properties of the silicon carbide are as follows: Vickers hardness (Hv) = 23 GPa, Young’s modulus (E) = 405 GPa, fracture toughness (K_IC_) = 2.9 MPa·m^1^/^2^, and a density of 3.1 g/cm^3^.

**Figure 1:**
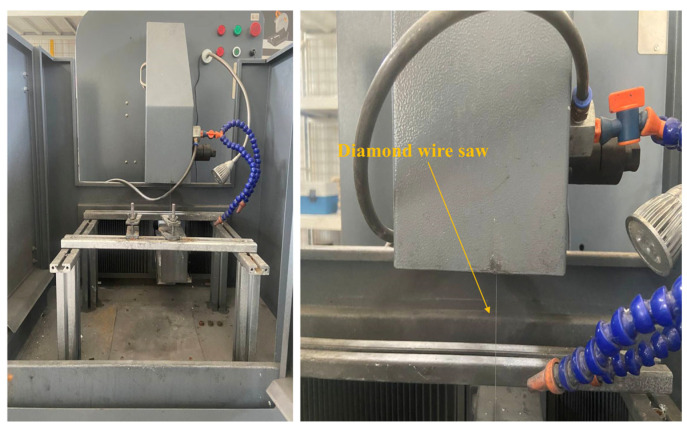
Experimental equipment apparatus.

The grinding experiments were conducted using a diamond wire-cutting machine (SH280YX, Shenghai Tec., Guangzhou, China), and shown in Figure 1. A diamond wire with an iron-based alloy wire core and diamond particles as the abrasive material was used, with a diameter of 0.38 mm. The diamond wire grinding was performed using a reciprocating grinding method. The grinding tests were carried out under the following conditions: wire speed of 30 m/s and feed rates of 0.25, 0.5, 1.0, and 2.0 mm/min. The appropriateness of the feed rate was assessed based on the vertical alignment of the wire during the cutting process. For this study, we selected a feed rate of 2.0 mm/min, which is 5 to 10 times higher than the rates commonly reported in the literature. To mitigate the risk of wire bending due to a slow removal rate, which significantly increases the normal force F in the feed direction and potentially impacts the experimental outcomes, we employed a wire speed of 30 m/s. During the grinding process, a water-based coolant was sprayed onto the grinding area from a nozzle to cool the diamond wire and silicon carbide material.

An X-ray diffractometer (D8 ADVANCE A25X, Bruker, Ettlingen, Germany) was used to analyze the phase composition of the silicon carbide ceramics. After the scratch tests, a field emission scanning electron microscope (FE-SEM) (Inspect F50, FEI, Hillsboro, OR, USA) was employed to observe the surface and chip characteristics. Additionally, focused ion beam (FIB) (Helios 5 CX, Thermoscientific, Waltham, MA, USA) technology was used to prepare samples from the scratch area, enabling a transmission electron microscope (ARM200F, JEOL, Tokyo, Japan) to analyze the microstructure and defect characteristics of the material. To accurately measure the scratches and surface morphology, a laser confocal microscope (VK-9700, KEYENCE, Osaka, Japan) was used for three-dimensional topography analysis, providing high-resolution surface morphology data and roughness information of the material post-grinding.

## 3. Results and Discussion

**Figure 2:**
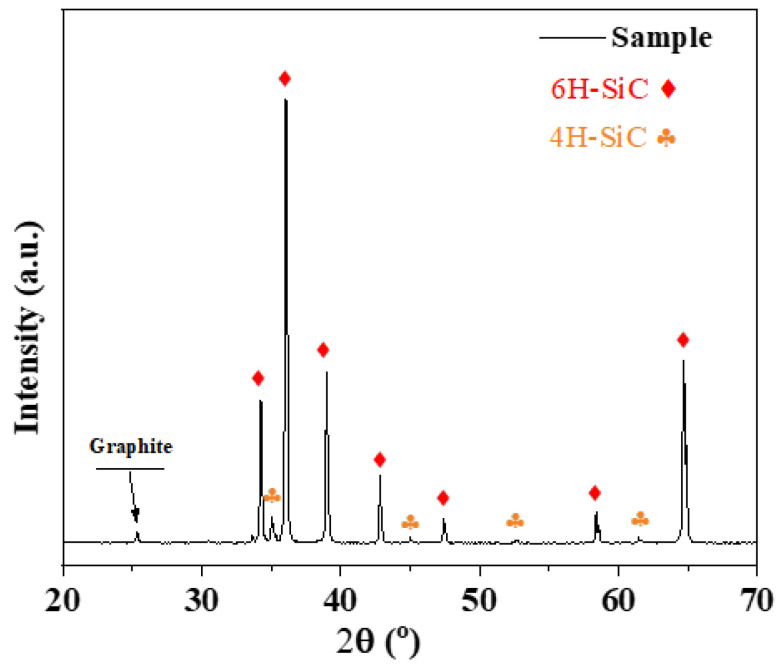
X-ray diffraction (XRD) pattern of as-obtained dense SiC ceramic.

Figure 2 shows the X-ray diffraction (XRD) pattern of the synthesized silicon carbide (SiC). By comparing with the International Centre for Diffraction Data (ICDD) database (https://www.icdd.com/; accessed on 17 November 2023), it can be observed that the three most substantial peaks of the sample match the data of PDF# 29-1131 and PDF# 29-1127. The results indicate that the main component of the sample is 6H-SiC with a small amount of 4H-SiC present. Based on the calculations, 6H-SiC accounts for approximately 94.23% of the sample, significantly higher than the content of 4H-SiC. A weak peak of graphite was observed in the XRD patterns, which is a general situation in SiC materials [28]. Therefore, it can be concluded that the 6H-SiC structure primarily influences the mechanical properties of the sample. Silicon carbide (SiC) commonly exhibits different crystal structures: the cube structure and the Silicon carbide (SiC) commonly exhibit various crystal structures, including the cubic (3C) and hexagonal forms. The hexagonal structure comprises 2H, 4H, and 6H-SiC. Research indicates that hexagonal structures generally offer superior mechanical properties compared to the cubic 3C structure [29]. Among the hexagonal forms, 4H-SiC and 6H-SiC display similar performance characteristics, but 6H-SiC typically shows slightly greater hardness and toughness than 4H-SiC [30]. This enhanced toughness of 6H-SiC contributes to better resistance against crack formation during processing. During sintering, the coexistence of 4H and 6H phases is unavoidable, and their relative proportions can directly affect processing quality. Thus, in studies of polycrystalline SiC, the structural composition of the material is critical. In this study, the polycrystalline SiC used has a high proportion of 6H-SiC. Consequently, the mechanical properties of the sample during grinding are mainly determined by 6H-SiC.

**Figure 3:**
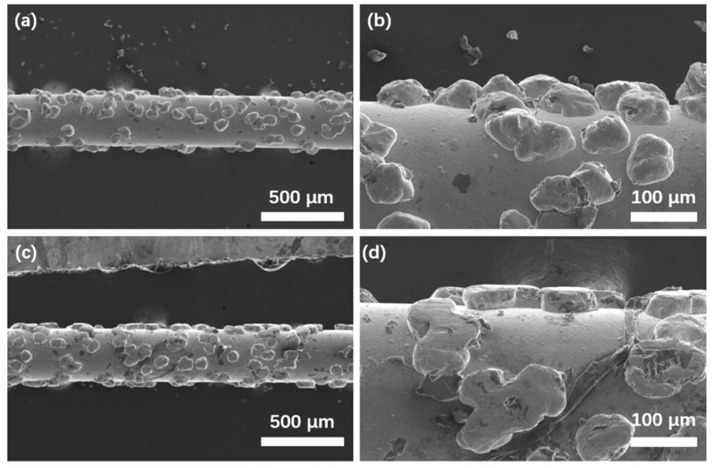
Scanning electron microscopy (SEM) images of diamond wire saw: (**a**,**b**) the new diamond wire; (**c**,**d**) the used diamond wire saw.

As a commonly used material removal method, this study employs diamond wire sawing. Diamond wire uses diamond particles as the grinding medium, differing from traditional grinding wheels that use surface contact. In diamond wire, all particles have an equal probability of participating in the grinding process. Therefore, scanning electron microscopy (SEM) can be used to characterize a segment of the diamond wire to observe the condition of the diamond grains before and after use. Figure 3a,b show the SEM images of the new diamond wire. The photos reveal that the diamond particles are uniformly distributed on the metal wire substrate, presenting a hill-like shape with distinct edges. At this stage, the morphology of the diamond particles is consistent with the abrasive grain morphology depicted in simulations. During grinding, the abrasive grains have an appropriate penetration angle, which effectively grinds the sample. Figure 3c,d displayed the SEM images of the used diamond wire. The images indicate that the number of abrasive grains has not significantly changed, demonstrating a good bond between the diamond grains and the substrate. It suggests that the non-rigid grinding method of diamond wire sawing does not easily cause the abrasive grains to fall off. On the other hand, all diamond grains have become flattened, and their heights are uniform after grinding, indicating that the abrasive grains themselves also undergo wear during the grinding process. Therefore, the role of abrasive grains in the grinding process is dynamically changing, and actual grinding phenomena differ from simulation results to some extent.

**Figure 4:**
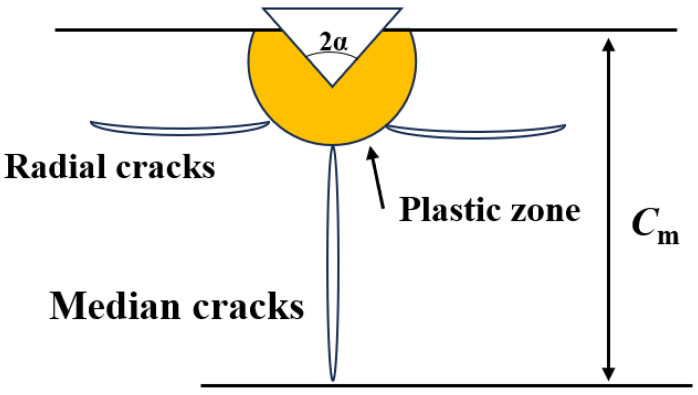
Schematic of typical crack systems in brittle materials.

According to indentation fracture mechanics studies, the grinding process can be considered as the phenomenon of abrasive particles creating localized indentations in brittle ceramics. Figure 4 is a schematic diagram of typical cracks in brittle materials, where the cross-sectional area can be divided into the plastic zone, radial crack zone, and central crack zone. Radial and central cracks play crucial roles in the grinding removal of brittle ceramics [31]. When materials are removed in a brittle manner, various processing factors can lead to the formation of radial and median cracks. Among these, radial cracks usually extend in the radial direction with little effect on the surface quality and mechanical properties of the material. Median cracks, which predominantly occur in the subsurface of the material and tend to be longer, can significantly impact the strength of the material. One of the factors influencing crack length is the abrasive grains used in the grinding process. The crack depth *C_m_* is related to the material properties, abrasive grain geometry, and abrasive material and can be expressed by the following equation [32]:

(1) $$C_{m}=0.206\frac{{\left(E\cdot H_{s}\right)}^{\frac{1}{3}}}{{\left(K_{c}\cdot \beta \right)}^{\frac{2}{3}}}{\left(\cot\alpha \right)}^{\frac{4}{9}}{\left(\tan\alpha \right)}^{\frac{4}{3}}\cdot {\left(h_{i}\right)}^{\frac{4}{3}}$$

where *E* is the elastic modulus, *H_s_* is the scratch hardness, *K_c_* is the fracture toughness, and *β* is a material-related parameter. From Equation (1), it can be observed that the elastic modulus, scratch hardness, fracture toughness, and *β* are parameters related to the material itself and do not change significantly during the grinding process. However, *α* and $h_{i}$, which are related to the abrasive grain geometry, change as the abrasive grains wear down, subsequently affecting *C_m_*. Among these, the most significant change occurs in the abrasive grain angle *α*, which transforms from a sharp protrusion to a horizontal step. As the abrasive grains wear down, the value of *α* gradually increases, leading to an increase in the depth of *C_m_*.

**Figure 5:**
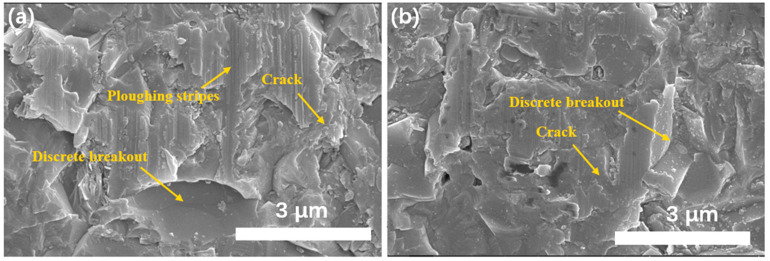
SEM images of SiC surface cutting by new diamond wire (**a**) and used diamond wire (**b**).

Figure 5a shows the SEM image of the SiC surface cut by the new diamond wire, and Figure 5b shows the SEM image of the SiC surface cut by the used diamond wire. Under the same wire speed, feed rate, and sample thickness conditions, the grinding surface in Figure 5a displays distinct plow marks, whereas the plow marks are significantly reduced in Figure 5b. It indicates that the change in the morphology of the diamond abrasive grains affects the grinding process to a certain extent, which is visually manifested as differences in the surface morphology of the ground silicon carbide. In Figure 5b, the step of perforation fracture can be seen. The presence of plow furrows, i.e., plastic deformation, is not observed, which is due to the fact that the sharp corners of the grits are already worn out, and due to the low depth of grind required for plastic deformation, the already flattened grits are not able to provide a low depth of grind similar to that of the single-grain grinding test; therefore, grinding is mainly dominated by brittle fracture removal [33]. It is evident that the morphology of abrasive grains changes dynamically during the grinding process. These changes negatively impact the control of the surface quality. To quantify this effect more precisely, further detailed research is required, such as molecular dynamics (MD) simulations.

**Figure 6:**
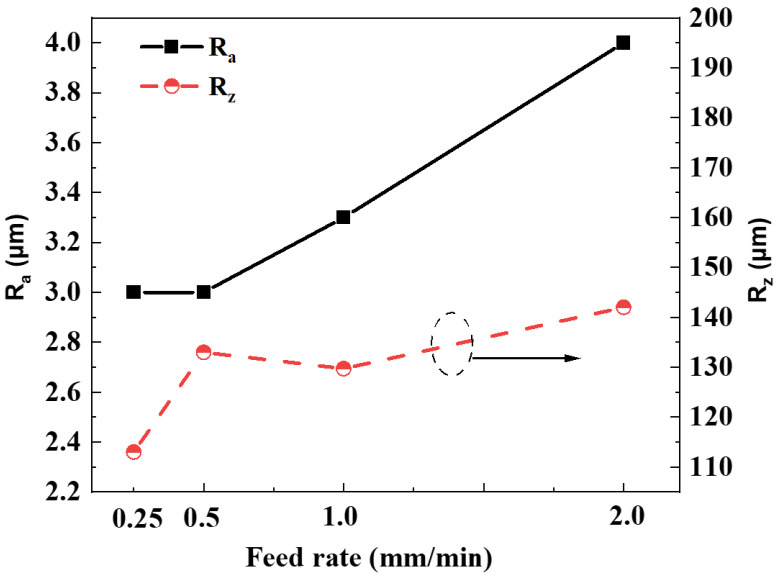
Surface roughness of machined SiC ceramic versus feed rate: the values of *R*_z_ is presented by the dotted line.

Figure 6 shows the relationship between different feed rates and the value of surface roughness *R*_a_ and *R*_z_. *R*_a_ (Arithmetic Average Roughness) measures the average deviation of surface heights from the mean line, providing a general assessment of surface texture. *R*_z_ (Average Maximum Height of the Profile) measures the average vertical distance between the highest peaks and lowest valleys within a sample, reflecting the total height of surface irregularities. Unlike fine grinding wheel polishing, diamond wire cutting is mainly used for cutting, and the grinding line reciprocates, so the roughness (*R*_a_) of the grinding surface is larger than that of the grinding wheel. The figure shows that there is no significant difference in the surface roughness (*R*_a_) values at lower grinding speeds (0.25 and 0.50 mm/min); with the increase in feed rate, the roughness gradually increases. When the feed rate reaches 2.0 mm/min, the surface roughness (*R*_a_) increases rapidly. The figure demonstrates that as the feed rate increases, the surface roughness (*R*_z_) also tends to rise. This value of *R*_z_ could be attributed to the faster speed of the abrasive wire, which creates pits during the grinding process. Overall, an increase in feed rate results in a decline in the quality of the ground surface. Therefore, when selecting specific process parameters, it is crucial to balance the quality of machining with efficiency to determine the optimal feed rate.

**Figure 7:**
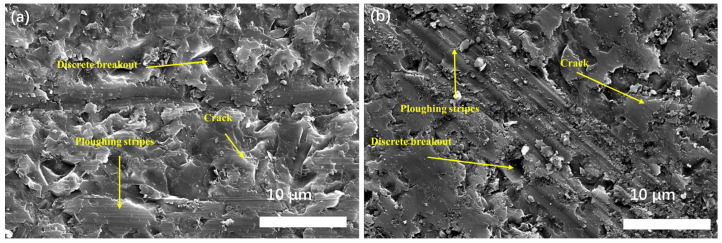
SEM images of the machined surface of SiC ceramics cut at the different feed rates: (**a**) 2.0 mm/min; (**b**) 0.25 mm/min.

In Figure 7, it can be seen that the silicon carbide surface under the grinding action of the diamond abrasive line shows a clear composite removal mechanism in which fracture removal and plastic removal meet. Plasticity traces along the grinding direction can be seen in the figure, and the grooves where the grains were pulled out by fracture can be seen around the plasticity traces. The presence of abrasive chips, which are also involved in the grinding of silicon carbide, can also be observed. The grinding process applies forces parallel and perpendicular to the grinding direction on the material surface. The force parallel to the grinding direction mainly plays a role in stripping the silicon carbide grains. In contrast, the force perpendicular to the silicon carbide surface will make the grains extend cracks along the direction of grinding, leading to grain breakage and eventual removal.

**Figure 8:**
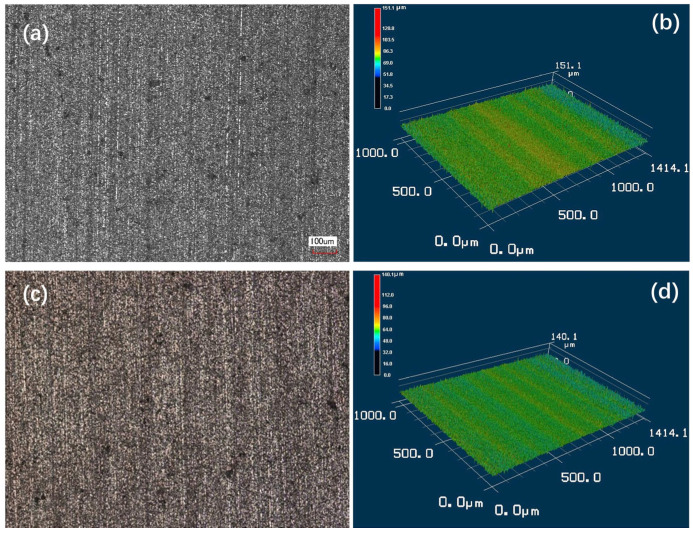
Laser confocal and contour maps of SiC ceramic samples cut at the different feed rates: (**a**,**b**) 2.0 mm/min; (**c**,**d**) 0.25 mm/min.

Typically, the feed rate significantly affects the quality of the ground surface. In this experiment, the test material was cut at feed rates of 2.0 mm/min and 0.25 mm/min in Figure 8. As the diamond wire used in this round of tests had not been subjected to prolonged grinding operations, clear plow marks were observed on the sample surfaces. Additionally, the sample surface ground at a feed rate of 2.0 mm/min exhibited more grain fracture and pullout morphology, while the sample surface ground at 0.25 mm/min was relatively smoother. This is due to the fact that the generated chips also participate during the grinding process. A slower feed rate allows more time for the chips to participate in grinding, thereby positively impacting the surface quality. Analysis of the ground surface using laser confocal microscopy further verified this phenomenon. The results showed that the sample surface ground at a feed rate of 0.25 mm/min was smoother with a significantly lower roughness than the sample surface ground at a feed rate of 2.0 mm/min. This indicates that a slower feed rate not only helps to reduce grain fracture and pullout but also significantly improves surface smoothness and finish.

The grinding removal process for brittle materials can be regarded as a simultaneous occurrence of plastic removal and brittle fracture removal. As shown in Figure 6, when the feed rate is slow (0.5 mm/min), the Ra (surface roughness) changes insignificantly, indicating a state of equilibrium. As the feed rate further increases, the balance between plastic removal and brittle fracture shifts, with brittle fracture gradually becoming dominant, leading to noticeable changes in surface quality. In the study of grinding Si_3_N_4_, it was observed that Ra decreases progressively with increasing cutting speed. Beyond 1200 m/min, Ra levels off until reaching 1600 m/min (with this study employing a speed of 1800 m/min) [24]. Therefore, by increasing the feed rate and cutting speed, it is possible to effectively balance the removal rate and surface quality in grinding processes.

Previous research has preliminarily identified that on a micron scale, diamond grinding removes silicon carbide ceramics through grain fracture and pullout. Additionally, it has been observed that surface grinding quality is related to process parameters. However, two main questions remain unresolved: (1) What changes occur within the grains and at the grain boundaries during the grinding process? (2) How does diamond grinding affect the internal grains of polycrystalline silicon carbide ceramics? A commonly accepted theory in the study of silicon carbide grinding mechanisms is the “ductile-to-brittle transition” theory. This theory suggests that when diamond abrasive grains first come into contact with the silicon carbide grains, plastic deformation occurs. As grinding progresses, brittle fracture of the silicon carbide grains ensues, leading to material removal. This method, primarily studied using single diamond particle scratching, differs from actual processing conditions. Therefore, it is necessary to investigate the grinding mechanisms of diamond wire saws under practical conditions. Focused Ion Beam (FIB) combined with Transmission Electron Microscopy (TEM) enables precise, site-specific sample preparation, producing lamellae typically less than 50 nm in thickness. This method is particularly suited for high-resolution analysis, allowing detailed observation of atomic structures in localized regions such as grain boundaries and defects, while preserving the material’s integrity. In this study, samples with clear grinding traces were sectioned perpendicular to the scratch direction using FIB to a depth of 5 μm and a thickness of 30 nm, then analyzed using TEM to study the internal grinding mechanisms of silicon carbide.

**Figure 9:**
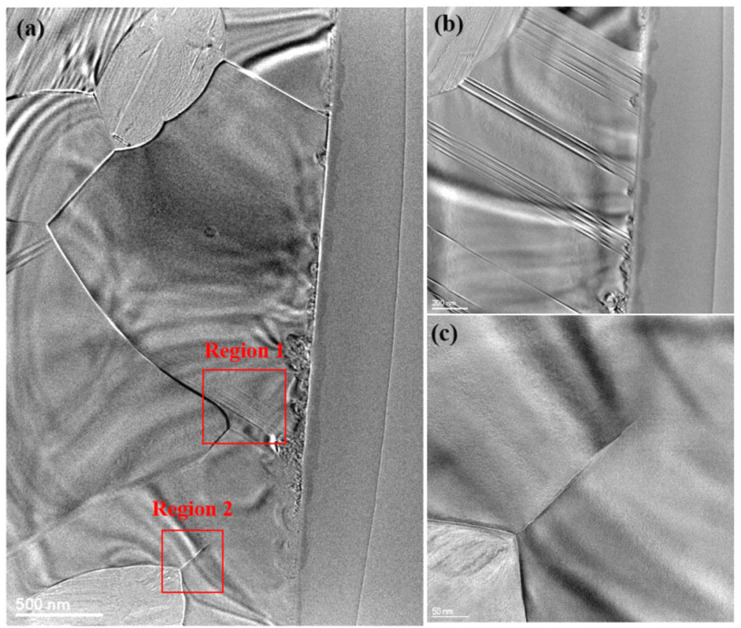
(**a**) Transmission electron microscopy (TEM) image of the cross-section of the machined surface of SiC ceramic, and (**b**,**c**) the magnified images of regions 1 and 2 in Figure (**a**).

Figure 9a shows alternating light and dark stripes reflecting the internal stress distribution within the SiC ceramics. These stripes start from the grinding surface, extending into the grains and crossing grain boundaries, thus affecting neighboring grains that are not directly contacted by the abrasives. The presence of internal stress can affect the mechanical properties of SiC ceramics. Observations show that these stripes extend from the grinding surface into the grains and across grain boundaries, affecting adjacent grains not directly contacted by the abrasives. The presence of internal stress significantly impacts the mechanical properties of SiC ceramics, potentially reducing material strength and durability. Figure 9b reveals numerous dislocation lines within the grains directly subjected to diamond grinding. Further grinding can lead to internal crack formation, eventually causing grain fracture. This demonstrates that the generation of dislocations in silicon carbide under diamond wire grinding is one of the removal mechanisms. Further grinding can induce internal cracks, leading to grain fracture. This confirms that dislocation generation under diamond wire grinding is a crucial removal mechanism for silicon carbide, indicating that dislocation formation and propagation are primary material removal methods during grinding. Figure 9c shows microcracks, which play a significant role in further grain fracture and removal during grinding and in crack propagation during use. The stress from grinding concentrates at grain boundaries, forming microcracks, which are crucial for further grain fracture and removal. Additionally, these microcracks may propagate during use, affecting overall material performance and lifespan. The existence and propagation of microcracks impact grinding efficiency and significantly influence the material’s performance and reliability.

**Figure 10:**
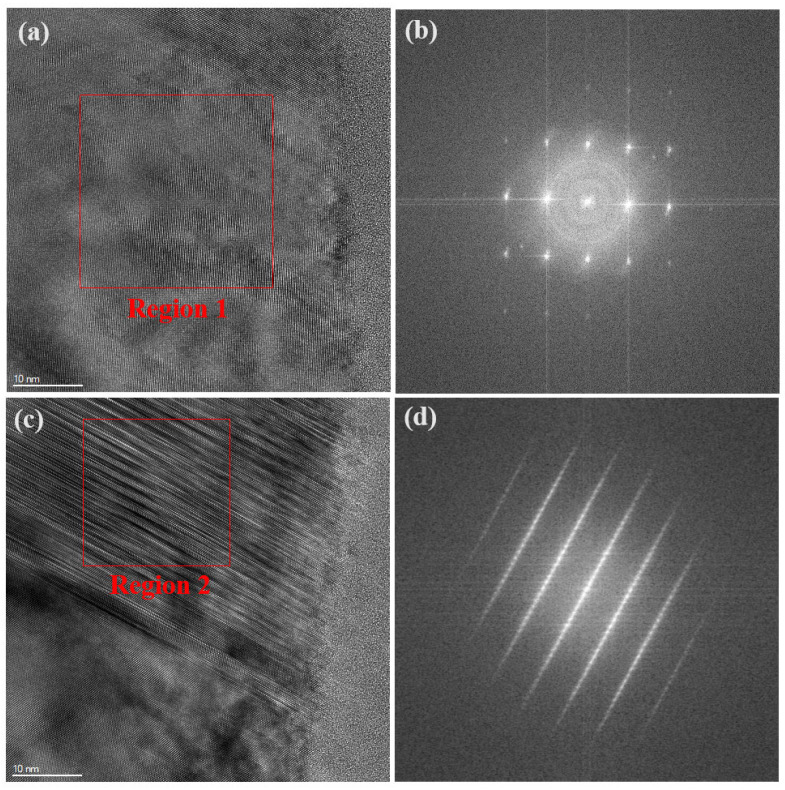
High-resolution transmission electron microscopy (HRTEM) images. (**a**,**c**) the typical images of machined SiC ceramic, and corresponding FFT results: (**b**) region 1 and (**d**) region.

Figure 10 presents high-resolution transmission electron microscopy (HRTEM) images and selected area electron diffraction (SAED) patterns of the internal region of silicon carbide grains subjected to diamond grinding. In Figure 10a, non-periodic areas can be observed within the grains. Fast Fourier Transform (FFT) analysis of this region yields the SAED pattern shown in Figure 9b, which further illustrates the presence of amorphous structures in this region [27]. This amorphization indicates that the crystal structure was severely disrupted in localized areas during grinding, forming an amorphous structure. In Figure 10c, numerous stacking faults are observable. FFT analysis of this area, as shown in the SAED image in Figure 10d, reveals the presence of reciprocal rods, further confirming the existence of stacking faults within this region. The presence of stacking faults indicates a misalignment in the local crystal structure, significantly impacting the material’s mechanical properties. Both amorphization and stacking faults disrupt the crystalline structure of silicon carbide, leading to grain fragmentation.

To summarize, the diamond wire saw removal mechanism for silicon carbide primarily involves factors such as crack formation, dislocation generation, and amorphization under external forces. These factors collectively contribute to the breakdown of the silicon carbide structure, leading to grain fragmentation. Ultimately, these mechanisms cause both transgranular and intergranular fractures, enabling effective grinding of silicon carbide. These findings are crucial for understanding the behavior of silicon carbide materials during the grinding process and for improving grinding techniques, thereby enhancing material processing quality and performance.

## 4. Conclusions

This study investigates the factors and removal mechanisms involved in diamond wire sawing of polycrystalline silicon carbide ceramics under high feed rate and high wire speed conditions. The results reveal that the coexistence of multiple crystal structures in silicon carbide influences the grinding removal process, with changes in the morphology of abrasive and feed rate impacting grinding efficiency. An initial analysis of the crystal structure composition, with a focus on 6H-SiC, is crucial. As the diamond wire is used, the morphology of the diamond abrasives evolves, leading to variations in the ratio of plastic to brittle removal and affecting surface quality. Under a wire speed of 30 m/s, surface roughness increases with feed rates exceeding 0.5 mm/min, offering insights into balancing processing quality and efficiency. Additionally, using a combination of FIB-TEM techniques, the study explored the removal mechanisms, revealing phenomena such as microcrack propagation, amorphous regions, and dislocations. These findings confirm that the removal mechanism of the diamond wire saw involves both plastic and brittle removal processes. This study provides a basis for further optimization of the process, including wire use and process parameters, to achieve efficient and high-quality removal of polycrystalline silicon carbide ceramics.

## Data Availability

The raw data supporting the conclusions of this article will be made available by the authors on request.
